# Molecular Mechanisms of Foot-and-Mouth Disease Virus Targeting the Host Antiviral Response

**DOI:** 10.3389/fcimb.2017.00252

**Published:** 2017-06-13

**Authors:** Miguel Rodríguez Pulido, Margarita Sáiz

**Affiliations:** Centro de Biología Molecular Severo Ochoa (Consejo Superior de Investigaciones Científicas-UAM)Madrid, Spain

**Keywords:** foot-and-mouth disease virus, innate immunity, viral evasion, interferon, viral RNA detection, virus-host interaction

## Abstract

Foot-and-mouth disease virus (FMDV) is the causative agent of an acute vesicular disease affecting pigs, cattle and other domestic, and wild animals worldwide. The aim of the host interferon (IFN) response is to limit viral replication and spread. Detection of the viral genome and products by specialized cellular sensors initiates a signaling cascade that leads to a rapid antiviral response involving the secretion of type I- and type III-IFNs and other antiviral cytokines with antiproliferative and immunomodulatory functions. During co-evolution with their hosts, viruses have acquired strategies to actively counteract host antiviral responses and the balance between innate response and viral antagonism may determine the outcome of disease and pathogenesis. FMDV proteases Lpro and 3C have been found to antagonize the host IFN response by a repertoire of mechanisms. Moreover, the putative role of other viral proteins in IFN antagonism is being recently unveiled, uncovering sophisticated immune evasion strategies different to those reported to date for other members of the *Picornaviridae* family. Here, we review the interplay between antiviral responses induced by FMDV infection and viral countermeasures to block them. Research on strategies used by viruses to modulate immunity will provide insights into the function of host pathways involved in defense against pathogens and will also lead to development of new therapeutic strategies to fight virus infections.

## Introduction

Foot-and-mouth disease virus (FMDV) is a member of the *Aphthovirus* genus in the *Picornaviridae* family and the etiologic agent of a highly infectious disease considered a major concern in animal health (Saiz et al., 2002; Knight-Jones et al., 2016). Disease control involves slaughter of infected and in-contact animals, restriction of animal movement, and vaccination based on chemically-inactivated virus (Robinson et al., 2016a). To develop new strategies for rapid FMDV control, including antiviral approaches and novel vaccines, it is essential to gain a better understanding of the virus-host interplay. FMDV interaction with lymphocytes, dendritic cells, and natural killer cells in swine and cattle has been previously reviewed (Golde et al., 2008; Grubman et al., 2008; Summerfield et al., 2009; Toka and Golde, 2013). On the front line of antiviral immunity is the rapid induction of type-I interferon (IFN) and other antiviral cytokines at the site of infection. Evasion of the host immune response may contribute to viral pathogenicity and viruses often display redundant strategies to counteract it (Zinzula and Tramontano, 2013; Fensterl et al., 2015). IFN-based strategies have proved to be an efficient biotherapeutic approach against FMDV (Rodriguez-Pulido et al., 2011; Robinson et al., 2016b; Borrego et al., 2017), and the study of viral mechanisms interfering with immune functions has been a very active area of research over the last few years. This minireview summarizes our current knowledge on how FMDV antagonizes the IFN-α/β induction and signaling pathways to circumvent the host antiviral response.

## Sensing of viral genome and induction of innate responses

**Figure 1:**
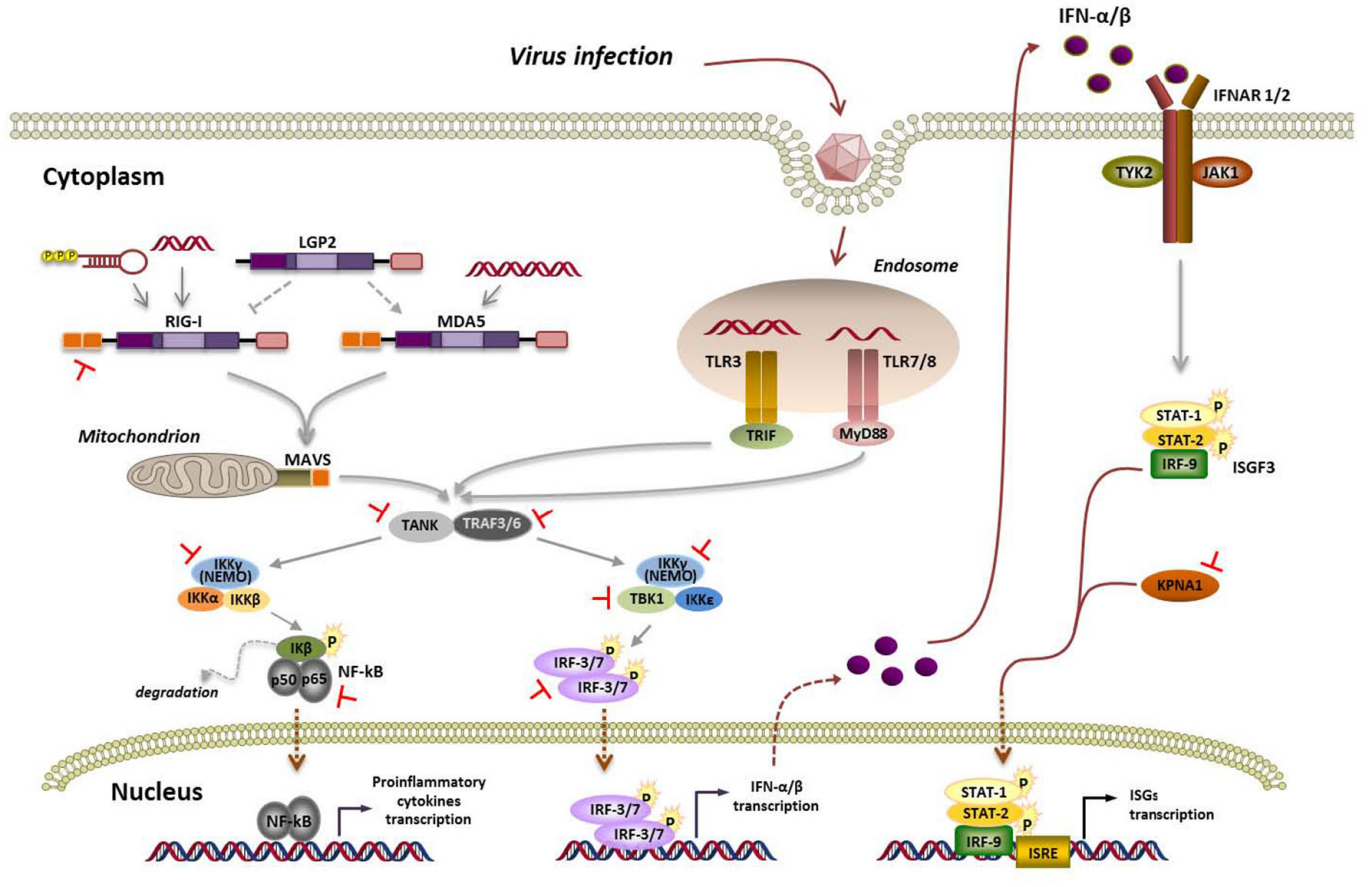
Viral RNA detection by RLRs and TLRs, signaling pathways and overview of FMDV strategies aimed at its supression. Interaction of viral RNA with nucleic acid sensing endosomal TLRs or cytosolic RLRs triggers a signaling cascade leading to the establishment of an antiviral state based on type-I IFN and proinflammatory cytokines induction. TLR7/8 and TLR3 signal through MyD88 (myeloid differentiation primary response 88) and TRIF (TIR-domain containing adaptor inducing IFN-β), respectively. When the inactive forms of RIG-I or MDA5 bind viral RNA, the helicases undergo ubiquitin-induced oligomerization and then interact with the adaptor molecule mitochondrial antiviral signaling protein (MAVS) through their caspase activation and recruiting domains (CARD). Activation of MyD88, TRIF, or MAVS triggers a signaling cascade including TRAF family member-associated NF-κB activator (TANK) and tumor necrosis factor (TNF) receptor-associated factor 3 and 6 (TRAF3/6). Then, two distinct pathways involving IKK-α/β/ɤ (IKK-ɤ also known as NEMO) and TBK1/IKK-ε are activated. TBK1 phosphorylates and activates the IFN-regulatory factors 3 and 7 (IRF-3/7) to induce the expression of type-I IFN genes, while the IKK-α/β/ɤ complex leads to activation of the nuclear transcription factor kappa B (NF-κB) and transcription of proinflammatory cytokines. The IFN response is then amplified and spread to surrounding uninfected cells by engagement of the IFN-α/β receptor (IFNAR) through the tyrosine kinases termed Janus kinases (JAKs) and their downstream transcription factors (signal transducers and activators of transcription, STATs), the JAK/STAT signaling cascade, leading to the expression of hundreds of IFN-stimulated genes (ISGs) containing IFN-stimulated response elements (ISREs) in their promoters. Blockade by FMDV at the different steps of the pathway are indicated in red.

Viral infections can stimulate multiple pathways to induce type-I and type-III IFNs which have antiviral, antiproliferative, and immunomodulatory functions (Fensterl et al., 2015). Maturation of dendritic cells (DC) is promoted by IFN-I, influencing the efficacy of the adaptive immune responses induced. IFN-α/β can be produced by virtually all nucleated cell types. The IFN response is then amplified and spread to surrounding uninfected cells by the expression of hundreds of IFN-stimulated genes (ISGs; Figure 1). Products encoded by ISGs together mediate the antiviral effect focused on degradation of viral nucleic acid or inhibition of viral gene expression. In most cases, type-I and type-III IFNs appear to be coproduced in response to viral infection, with similar mechanisms of induction, signal transduction, and biological activities (Uze and Monneron, 2007).

Recognition of viral pathogen-associated molecular patterns (PAMPs) in the invaders that are “non-self” to the cell by pattern-recognition receptors (PRRs) activates a signaling cascade leading to the expression of type-I IFN genes and proinflammatory cytokines (Figure 1). Among the PRRs involved in sensing RNA viral genomes, *Toll*-like receptors (TLRs), expressed on the surface and endosomal compartments of some cell types, and retinoic acid-inducible gene-I (RIG-I)-like receptors (RLRs), ubiquitous cytosolic RNA helicases, have a remarkable role (Bruns and Horvath, 2014; Lester and Li, 2014). Among TLRs linked to antiviral immunity, TLR3 recognizes double-stranded RNA (dsRNA), while TLR7 and TLR8 detect single-stranded RNA (ssRNA; Lund et al., 2004). The RLRs RIG-I and melanoma differentiation-associated gene 5 (MDA5) play critical roles in triggering immune defenses against RNA virus infection. RIG-I and MDA5 recognize distinct RNA species that have reached the cytoplasm by infection or transfection (Schlee, 2013). It is widely accepted that RIG-I ligands include ssRNA bearing a 5′-triphosphate and a blunt-ended region at the 5′end, as well as short dsRNA, while MDA5 binds to long dsRNA (Berke et al., 2013; Liu et al., 2016). While RIG-I is required for sensing, among others, paramyxoviruses, influenza virus, Japanese encephalitis virus, and hepatitis C virus, MDA5 is involved in recognizing picornaviruses, mouse norovirus, mouse hepatitis virus and defective interfering particles of paramyxoviruses (Feng et al., 2014).

LGP2 (Laboratory of Genetics and Physiology 2) is the third member of the RLR family. As LGP2 lacks the N-terminal caspase activation and recruiting domains (CARDs) used by RIG-I and MDA5 for downstream signaling (Figure 1), its role in antiviral response is still controversial. Indeed, opposing roles of LGP2 as both a positive or negative regulator have been reported. Titration experiments of overexpressed LGP2 showed an enhancing effect on MDA5-mediated signaling when LGP2 was present at low cellular concentrations. At high LGP2 concentrations, inhibition of MDA5 signaling was observed. The authors proposed that LGP2 regulatory role works as a concentration dependent biphasic switch, enhancing MDA5-mediated antiviral signaling at early stages of infection when LGP2 concentration is low, but as infection progresses and IFN induces LGP2 production, MDA5 signaling is inhibited (Bruns et al., 2013, 2014; Uchikawa et al., 2016).

The dsRNA synthesized during picornavirus replication has been found to activate MDA5 (Feng et al., 2012). Interestingly, a picornavirus-derived MDA5 agonist was found through its interaction with LGP2 (Deddouche et al., 2014). LGP2/RNA complexes purified from cells infected with encephalomyocarditis virus (EMCV) contained a specific sequence corresponding to the L region of the EMCV antisense RNA with strong MDA5-stimulatory activity.

Using lentivirus-driven RNA interference, Husser et al. reported that FMDV was sensed by MDA5 but not by RIG-I or TLR3 in porcine epithelial cells. Six hours after FMDV infection, IFN-β mRNA induction was only reduced significantly in MDA5 silenced porcine kidney (PK-15) cells (Husser et al., 2011). These results indicate that IFN-β transcription is triggered in an MDA5-dependent manner regardless of the inhibition of host translation and other specific antagonistic actions exerted by the virus which normally result in a failed response against FMDV infection. In plasmacytoid dendritic cells (pDC), FMDV seems to be sensed by TLR7 inside the endosomal compartment (Guzylack-Piriou et al., 2006; Lannes et al., 2012). The relevance of the IFN-α/β induced double-stranded RNA-dependent protein kinase R (PKR) in the inhibition of FMDV replication has been well-documented in swine and bovine cells (Chinsangaram et al., 2001; de Los Santos et al., 2006). Treatment with an inhibitor of PKR increased the virus yield for several folds compared with that of the untreated infected cells and PKR down-regulation by RNA interference also resulted in higher viral titers, further confirming a direct function of PKR in controlling FMDV replication.

Experiments in pigs showed a good correlation between IFN-induced protection against FMDV and induction of ISGs in skin, peripheral blood mononuclear cells, and lymphoid tissues (Diaz-San Segundo et al., 2010). The role of an enzyme involved in epigenetics modifications, the histone methyltransferase EHMT2, in regulation of bovine IFN-β gene expression and in the establishment of an antiviral state in primary bovine fibroblast cells has been reported, as well as the prophylactic and therapeutic use of EHMT2 inhibitors to control FMDV replication (Singh et al., 2016).

## Strategies used by picornaviruses to circumvent innate responses

**Figure 2:**
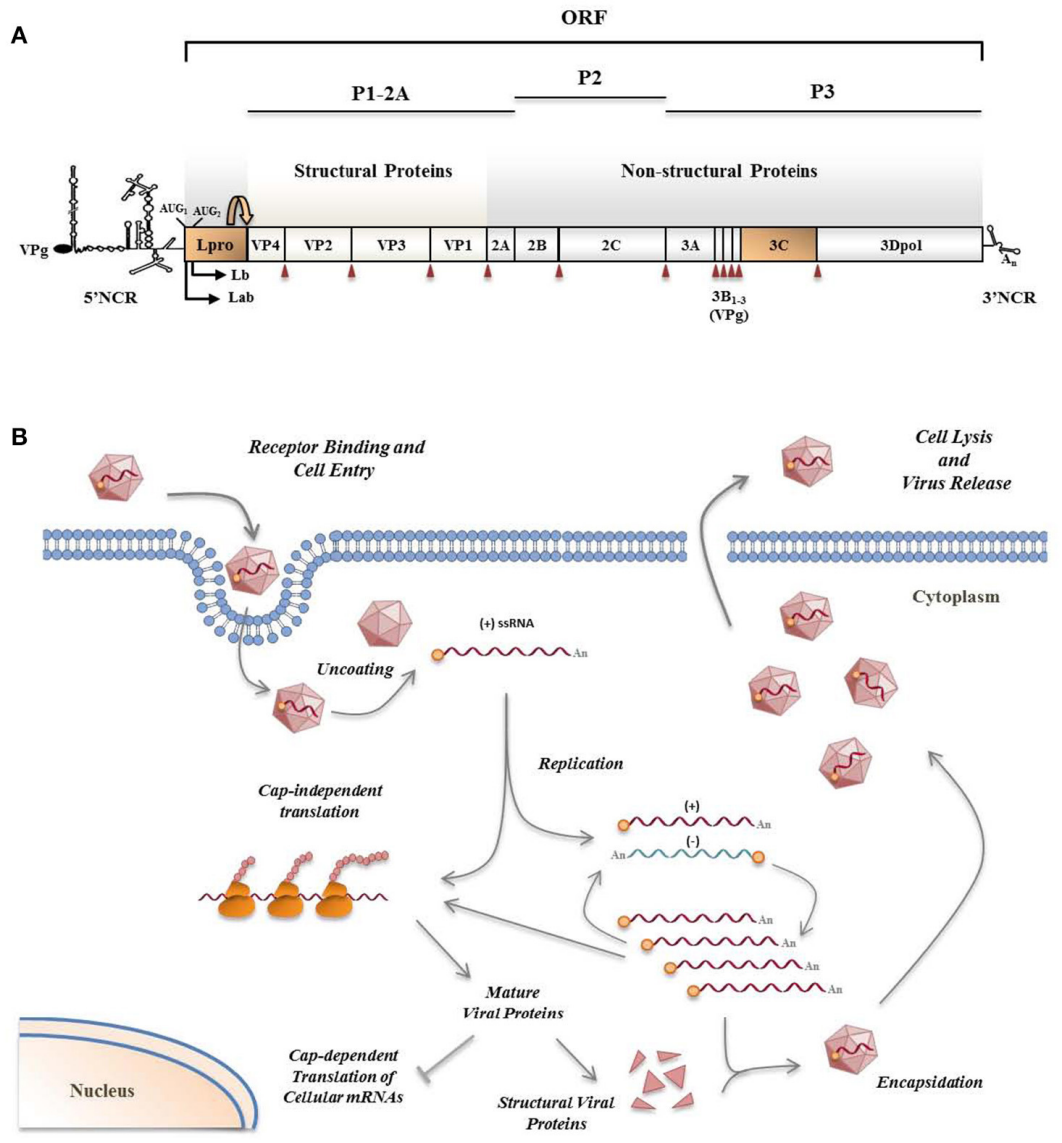
FMDV genome **(A)** and viral life cycle **(B)**. **(A)** FMDV has a positive sense ssRNA genome of about 8.5 Kb in length encoding a single polyprotein which undergoes proteolytic processing by viral proteases. Two highly structured non-coding regions flank the viral RNA which is poly-adenylated at its 3′-end and covalently linked to a viral protein (VPg) at its 5′-end. Primary co-translational processing of the polyprotein yields P1-2A, P2, and P3 precursors by the cleavage activity of the leader protease (Lpro), 3Cpro and a translational recoding event mediated by 2A. Lpro, a papain-like cysteine proteinase, is present as two different forms, Labpro and Lbpro, generated by translation initiation at two in-frame AUG codons separated by 84 nt on the viral RNA (Sangar et al., 1987) and subsequent intramolecular self-processing. The mature individual viral products include the four structural proteins—VP4, VP2, VP3, and VP1—, and 10 non-structural proteins—Lpro, 2A, 2B, 2C, 3A, 3B_1–3_ (three non-identical copies of VPg), 3Cpro and 3Dpol. 3Cpro cleavage sites are indicated by red arrowheads. **(B)** The entire replication cycle of the virus occurs in the cytoplasm. The viral RNA contains all the information required to take over the cellular machinery and induce the shut-off of the host macromolecular synthesis in infected cells where the viral products are translated by a cap-independent manner. VPg-primed RNA replication is carried out by the RNA-dependent RNA polymerase 3Dpol which transcribes the positive-strand RNA into a complementary, negative-strand RNA molecule. Then, 3Dpol generates multiple positive-strand RNAs which either enter a new round of translation and RNA replication, or are packaged by the capsid proteins to form new virus particles which are finally released by cell lysis.

The FMDV positive sense ssRNA encodes a polyprotein which is processed by the viral proteases in the cytoplasm of infected cells (Figure 2A). During the long co-evolution with their hosts, viruses have acquired strategies to actively counteract host antiviral responses, and picornaviruses, despite the limited size of their genomes, are no exception and have selected proteins serving multiple purposes maximizing virus replication and dissemination (Figure 2B). Besides their function in polyprotein processing, viral proteases target a variety of host proteins. Some viral proteases can inhibit type-I IFN production and NF-κB signaling through the cleavage of receptors, adaptors, and regulators participating in these pathways (Schulz and Mossman, 2016). Different strategies to evade the RLR-mediated type-I IFN response have been reported for picornaviruses, including FMDV, though most work focusses on the well-characterized genera *Enterovirus* and *Cardiovirus* (Dotzauer and Kraemer, 2012; Feng et al., 2014).

Picornavirus-induced host gene expression shut-off is known to contribute to IFN-α/β suppression though the primary blockade of this pathway lies upstream of IFN production. MDA5 and MAVS cleavage during enteroviral infections has been associated mainly to 2Apro, though cleavage of overexpressed MAVS by 3Cpro has also been shown (Feng et al., 2014). Interestingly, cleavage of RIG-I by 3Cpro has also been reported for various enteroviruses (Barral et al., 2009; Papon et al., 2009) though this molecule seems not involved in their recognition. Cardioviruses do not target MDA5 or MAVS but effectively prevent IRF-3 phosphorylation. The cardiovirus leader proteins have long been established as their main IFN antagonists. These multifunctional proteins lack enzymatic activity but interfere with critical cellular processes. They are known to disrupt the RanGDP/GTP gradient across the nuclear pore, likely interfering with IRF-3 nuclear translocation (Porter et al., 2006) and also to inhibit the activity of the IFN-induced RNase L by direct protein-protein interaction (Sorgeloos et al., 2013).

MAVS cleavage activity has been reported for 3ABC, a precursor of Hepatitis A virus (HAV, genus *Hepatovirus*) 3Cpro. The transmembrane domain in 3A targets 3ABC to the mitochondria outer membrane where MAVS is localized and cleaved (Yang et al., 2007).

Coxsackievirus B (CVB), enterovirus 71 (EV71) and HAV are also known to cleave the TLR3 adaptor TRIF in infected cells and 3Cpro has been linked to those cleavage events (Lei et al., 2011; Mukherjee et al., 2011; Qu et al., 2011). Recently, CVB and EMCV 3Cpro has been associated with degradation of Moloney leukemia virus 10, homolog (mouse; MOV10), an SF1 family RNA helicase involved in RNA silencing, translation and tumor suppression. MOV10 binds viral RNA promoting IFN induction by RIG-I/MAVS-independent pathway using IKK-ε and IRF3 as signaling mediators (Cuevas et al., 2016).

The role of exosomes in activation/evasion of the virus-induced innate immune response is being explored. These vesicles act as intercellular messengers to shuttle RNA and viral particles and are able to induce type-I IFN responses upon uptake by pDC. Paradoxically, some viruses like HAV have co-evolved to use exosomes to spread undetected by humoral immunity (Longatti, 2015).

## FMDV interference with innate antiviral responses

### Role of viral proteases

FMDV Leader (Lpro) and 3C proteases (Figure 2A) are known to have a relevant role on type-I IFN antagonism. Aphthovirus Lpro impairs cap-dependent translation through cleavage of initiation factor eIF4G, leading to a translational host shut-off (Devaney et al., 1988; Medina et al., 1993). Using an engineered FMDV lacking the Lpro coding region, de Los Santos et al. demonstrated that Lb inhibits the induction of IFN-β mRNA and the expression of ISGs in IBRS-2 swine cells (de Los Santos et al., 2006). This inhibitory effect was associated with Lpro translocation to the nucleus and degradation of the p65 subunit of NF-κB (de Los Santos et al., 2007; Zhu et al., 2010). Disruption of a predicted SAP (SAF-A/B, Acinus, and PIAS) domain between amino acids 47 and 83 of Lb selectively prevented p65/RelA processing without affecting eIF4G cleavage (de Los Santos et al., 2009). Microarray data showed that genes differentially expressed in primary bovine cells infected with FMDV leaderless vs. wild type viruses were involved in the innate immune response, being NF-κB primarily responsible for the observed changes (Zhu et al., 2010). Knockdown of cellular PKR by RNA interference did not show obvious effect on wild type virus yield, but resulted in a higher yield of the leaderless virus, suggesting a direct function of PKR to control FMDV replication in the natural host and the relationship of FMDV Lpro and PKR (de Los Santos et al., 2006). Lab and Lb have both been shown to suppress dsRNA-induced IFN-β mRNA transcription in PK-15 cells through decreasing IRF-3/7 expression (Wang et al., 2010).

Lpro has also been associated with regulation of the inflammatory and antiviral chemokine RANTES (regulated upon activation, normal T-cells expressed and secreted). Lpro limits RANTES and TNF-α transcription by degradation of p65/RelA (de Los Santos et al., 2007). Suppression of RANTES via ISRE by interfering with IRF-3/7 activation required catalytic activity and the SAP domain of Lpro (Wang et al., 2011a).

Sequence and structural bioinformatics analyses suggested similarities between the topology of FMDV Lbpro and that of some cellular and viral deubiquitylation enzymes (DUBs). Experimental evidence confirmed Lbpro as a novel viral DUB that cleaves ubiquitin moieties from critical signaling proteins of the type-I IFN signaling pathway, such as RIG-I, TBK1, TRAF3, and TRAF6 (Wang et al., 2011b; Figure 1). Mutations that ablate the catalytic activity or disrupt the SAP domain of Lbpro abrogated the DUB activity and blockade of IFN-β induction.

FMDV Lpro has been found to be an IFN-III antagonist in PK-15 cells. Recombinant porcine IFN-λ1 exhibited significant antiviral activity against FMDV but IFN-λ1 induction could not be detected in infected cells. Expression of Lpro inhibited poly(I:C)-induced IFN-λ1 promoter activity (Wang et al., 2011c). The catalytic activity and the SAP domain of Lpro were required for suppression.

Recently, the interaction of Lpro and the host transcription factor ADNP (activity dependent neuroprotective protein) has been detected by mass spectrometry (Medina et al., 2017). This interaction seems to be required for efficient viral replication. As ADNP is processed during FMDV infection and Lpro catalytic activity is required, the authors postulate that early post infection, Lpro may promote binding of ADNP, or other transcription factors of the same complex to specific promoter sequences, enhancing the repressive activity on IFN and ISGs transcription.

FMDV 3Cpro is a trypsin-like serine protease whose primary role is processing of the viral polyprotein (Figure 2A). However, 3Cpro also cleaves host proteins within infected cells, including eIF4G at a different site than Lpro and eIF4AI, likely contributing to the host shut-off (Li et al., 2001). Interestingly, FMDV 3C cleaves histone H3 during FMDV infection resulting in a generalized repression of cellular transcription (Falk et al., 1990). First evidence that FMDV 3Cpro is another virus-encoded IFN antagonist was provided when its ability to cleave NEMO (or Ikk-ɤ), an essential adaptor for IRFs and NF-κB activation (Figure 1), was reported (Wang et al., 2012). NEMO cleavage impairs its ability to activate downstream IFN production in the RLR and TLR pathways as the fragments resulting from 3Cpro cleavage were deficient in activation of NF-κB, IRF, and IFN induction. The cleavage of porcine NEMO in infected cells was also demonstrated. 3Cpro cleaved NEMO at the Gln 383 residue and catalytically inactive mutants failed to cleave NEMO (Wang et al., 2012).

FMDV has also been shown to inhibit the type-I IFN signaling pathway (Du et al., 2014). Expression of 3Cpro significantly reduced the transcript levels of ISGs and ISRE promoter activity. 3Cpro inhibits the JAK/STAT signaling pathway by blocking the nuclear translocation of STAT1/STAT2 (Figure 1). Phosphorylated STAT1 accumulates in the nucleus via interaction with a specific nuclear localization signal receptor, karyopherin α1 (KPNA1), whose degradation was specifically attributable to the protease activity of FMDV 3Cpro. Degradation of endogenous KPNA1 was also observed in PK-15 cells after FMDV infection (Du et al., 2014). This was the first example of a viral immune evasion mechanism involving direct degradation of KPNA1 by a viral protease, not by a proteasome- and caspase-dependent pathway.

The cleavage of TANK (Figure 1) by proteases encoded by several positive strand RNA viruses, including FMDV, has also been reported (Huang et al., 2015). TANK cleavage by EMCV 3C impairs its ability to inhibit TRAF6-mediated NF-κB signaling. FMDV 3C also cleaves TANK although at different cleavage sites, generating a 15 KDa N-terminal fragment. Cleavage was dependent on FMDV 3C protease activity. Expression of FMDV Lpro reduced the abundance of TANK, but no cleaved fragment was detected. Though the functions of the cleaved fragments under natural infections remain unknown, TANK cleavage by 3C may represent a common mechanism among positive strand RNA viruses to regulate NF-κB signaling interfering with host innate and inflammatory responses.

Suppression of autophagy induced at the early stages of infection via degradation of ATG5-ATG12 by FMDV 3Cpro has been recently reported (Fan et al., 2017). Replenishment of ATG5-ATG12 in PK-15 cells upregulated the expression of anti-viral proteins via recovery of NF-κB and IRF3 activation, thereby limiting FMDV replication. The CARDs of ATG5-ATG12 are known to associate with RIG-I and MAVS, regulating the innate immune response in a virus-dependent manner. Cleavage of ATGs by 3Cpro might also contribute to immune evasion hampering the delivery of viruses to lysosomes for degradation. However, previous work showed that autophagy may be beneficial for FMDV as virus yields were reduced in cells lacking ATG5, suggesting that FMDV induces autophagosomes during cell entry to facilitate infection (Berryman et al., 2012). Additionally, drug-induced autophagy in FMDV-infected cells increased viral yield, and inhibition of the autophagosomal pathway decreased viral replication (O'Donnell et al., 2011). Further studies will be required to understand the mechanism of autophagy induction and inhibition during FMDV infection and their role in the viral cycle.

### Role of other viral products

A few reports involve FMDV capsid proteins VP1 and VP3, as well as non-structural proteins 2B and 3A (Figure 2A), all of them lacking protease activity, in suppression of type-I IFN response to viral infection by not well-defined mechanisms.

VP1-induced suppression of type-I IFN response by its interaction with host cell protein sorcin has been reported (Li et al., 2013). VP1-sorcin interaction was detected by two-hybrid screening of a swine spleen cDNA library and immunoprecipitation assays. A reduction on the TNF-α or Sendai virus (SeV)-induced activation of IFN-α/β and NF-κB transcription was observed when VP1 was overexpressed in human HEK293T cells, both abolished after sorcin knockdown. The authors concluded that sorcin is required for VP1-induced suppression of type-I IFN response. How sorcin interferes with the IFN pathway or the biological relevance of the interaction remain unknown. Interestingly, studies in human cancer cell lines have shown that recombinant VP1 downregulates IKK/NF-κB and cyclooxygenase-2/prostaglandin E2 (COX-2/PGE2) signaling after binding to integrin receptors in target cells, which might be relevant for immune response pathways (Ho et al., 2014).

Counteracting of the type-II IFN (IFN-γ) signaling pathway by FMDV VP3 has been reported (Li et al., 2016b). Overexpression of VP3 in HEK293T inhibited the phosphorylation, dimerization and nuclear accumulation of STAT1, disrupting the assembly of the JAK1/STAT1 complex. VP3 interacted with endogenous JAK1 and adversely affected JAK1 protein levels. Lysosomal inhibitors blocked JAK1 degradation. The authors suggest a novel mechanism by which FMDV VP3 counteracts the type-II IFN signaling pathways inducing JAK1 degradation via a lysosomal pathway. The expression of FMDV VP3 has also been found to inhibit IRF3 phosphorylation and dimerization, as well as SeV-triggered activation of the IFN-β and ISRE promoters (Li et al., 2016c). The FMDV genome copies were increased in VP3-transfected PK-15 cells. These authors, however, did not detect any antagonistic effect by FMDV VP1 or P1 overexpression. In transient transfection and co-immunoprecipitation experiments, they found interaction between FMDV VP3 and MAVS by their corresponding C-terminal domains. Interaction with endogenous MAVS and a decrease in its expression could be also detected. They suggest that other proteins could mediate in the observed VP3-induced inhibition of MAVS mRNA (Li et al., 2016c).

FMDV 2B is a non-structural protein involved in the rearrangement of host cell membranes and disruption of the cellular secretory pathway (Moffat et al., 2007) that acts as a viroporin inducing autophagy and enhancing virus release (Ao et al., 2015). Zhu et al. observed that RIG-I protein was gradually downregulated as infection progresses (Zhu et al., 2016). Expression of RIG-I inhibited FMDV replication in a dose-dependent manner whereas an enhancement was observed in RIG-I siRNA PK-15 cells. The decrease of RIG-I was independent of eIF4G cleavage and apoptosis, proteasome, lysosome and caspase pathways. Direct interaction of 2B and RIG-I was detected in HEK293T cells and the 2B regions involved were determined. The 2B-induced reduction of RIG-I was independent of its viroporin activity. 2B did not induce reduction of MAVS, TBK1, or IRF3. The authors suggest that 2B interaction with RIG-I might recruit other proteins in a complex to degrade RIG-I by uncharacterized mechanisms. The same group has recently described the inhibition of LGP2 expression during FMDV infection and suggests the involvement of 2B by direct interaction with LGP2 (Zhu et al., 2017).

FMDV 3A is a multifunctional non-structural protein that plays important roles in virus replication, virulence and host-range determination (Gonzalez-Magaldi et al., 2014). 3A protein has been identified as a negative regulator of virus-triggered IFN-β signaling pathway (Li et al., 2016a). Overexpression of 3A inhibited SeV-triggered activation of IRF3 and the expression of RIG-I/MDA5 in HEK293T and PK15 cells. Transfection and co-immunoprecipitation experiments suggested that 3A interacts through its N-terminal region with RIG-I, MDA5, and MAVS. Disruption of the RIG-I, MDA5, and MAVS mRNA levels was observed when 3A was overexpressed.

For accurate interpretation of the results summarized above, it should be mentioned that capsid proteins are not produced individually during infection but generated by cleavage of pre-folded precursors and remaining associated with the other capsid proteins. 2B and 3A proteins are also produced from precursors detectable in infected cells. For that reason, the structure and function of the individually expressed proteins may not resemble the real situation during FMDV infection.

## Conclusions and outstanding questions

The accumulated information illustrates the intricate interaction of FMDV with host defenses. The virus has evolved a wide variety of strategies for targeting innate immunity components by the viral-encoded proteases. However, it might represent the tip of the iceberg as new viral products are increasingly being found by some means involved in immune evasion. Though in most cases the underlying mechanisms still need to be elucidated, likely novel strategies of viral antagonism will shortly be uncovered. More in-depth understanding of how FMDV-derived proteins lacking protease activity can impact and regulate the IFN signaling pathway is needed, as a better understanding on why the virus might target RIG-I or other factors apparently not directly involved in its sensing. Further studies should be focused on addressing the biological significance of the antagonistic events under physiological conditions of FMDV infection and preferably conducted in primary cells from relevant target tissues of natural host species. The cellular and molecular mechanisms operating in FMDV proteins-host interactions await future investigation and exciting results.

## Author contributions

All the authors listed have made substantial, direct, and intellectual contribution to the work, and approved it for publication.

### Conflict of interest statement

The authors declare that the research was conducted in the absence of any commercial or financial relationships that could be construed as a potential conflict of interest.
